# Leveraging AI and patient metadata to develop a novel risk score for skin cancer detection

**DOI:** 10.1038/s41598-024-71244-2

**Published:** 2024-09-06

**Authors:** Shafiqul Islam, Gordon C. Wishart, Joseph Walls, Per Hall, Alba G. Seco de Herrera, John Q. Gan, Haider Raza

**Affiliations:** 1 School of Computer Science and Electronic Engineering, University of Essex, Colchester, UK; 2 Check4Cancer Ltd., Cambridge, UK; 3 School of Medicine, Anglia Ruskin University, Chelmsford, UK; 4 Fitzwilliam Hospital, Peterborough, UK; 5 Addenbrookes Hospital NHS Foundation Trust, Cambridge, UK

**Keywords:** Skin cancer, Predictive markers, Risk factors, Scientific data, Computer science, Information technology

## Abstract

Melanoma of the skin is the 17th most common cancer worldwide. Early detection of suspicious skin lesions (melanoma) can increase 5-year survival rates by 20%. The 7-point checklist (7PCL) has been extensively used to suggest urgent referrals for patients with a possible melanoma. However, the 7PCL method only considers seven meta-features to calculate a risk score and is only relevant for patients with suspected melanoma. There are limited studies on the extensive use of patient metadata for the detection of all skin cancer subtypes. This study investigates artificial intelligence (AI) models that utilise patient metadata consisting of 23 attributes for suspicious skin lesion detection. We have identified a new set of most important risk factors, namely “*C4C risk factors*”, which is not just for melanoma, but for all types of skin cancer. The performance of the C4C risk factors for suspicious skin lesion detection is compared to that of the 7PCL and the Williams risk factors that predict the lifetime risk of melanoma. Our proposed AI framework ensembles five machine learning models and identifies seven new skin cancer risk factors: lesion pink, lesion size, lesion colour, lesion inflamed, lesion shape, lesion age, and natural hair colour, which achieved a sensitivity of $80.46\pm 2.50\%$ and a specificity of $62.09\pm 1.90\%$ in detecting suspicious skin lesions when evaluated using the metadata of 53,601 skin lesions collected from different skin cancer diagnostic clinics across the UK, significantly outperforming the 7PCL-based method (sensitivity $68.09\pm 2.10\%$, specificity $61.07\pm 0.90\%$) and the Williams risk factors (sensitivity $66.32\pm 1.90\%$, specificity $61.71\pm 0.6\%$). Furthermore, through weighting the seven new risk factors we came up with a new risk score, namely “*C4C risk score*”, which alone achieved a sensitivity of $76.09\pm 1.20\%$ and a specificity of $61.71\pm 0.50\%$, significantly outperforming the 7PCL-based risk score (sensitivity $73.91\pm 1.10\%$, specificity $49.49\pm 0.50\%$) and the Williams risk score (sensitivity $60.68\pm 1.30\%$, specificity $60.87\pm 0.80\%$). Finally, fusing the *C4C risk factors* with the 7PCL and Williams risk factors achieved the best performance, with a sensitivity of $85.24\pm 2.20\%$ and a specificity of $61.12\pm 0.90\%$. We believe that fusing these newly found risk factors and new risk score with image data will further boost the AI model performance for suspicious skin lesion detection. Hence, the new set of skin cancer risk factors has the potential to be used to modify current skin cancer referral guidelines for all skin cancer subtypes, including melanoma.

## Introduction

Malignant melanoma is solely responsible for 80% of all skin cancer deaths^1^. Delays in early detection of melanoma decrease 5-year survival rates by 20% as reported in the previous study^2^, which included the United Kingdom (UK) population. The UK follows a 2-week wait pathway system, where skin lesions suspicious of melanoma or squamous cell carcinoma (SCC) are seen by a specialist within 2 weeks. The referrals for this 2-week pathway have increased dramatically in recent years (159,430 patients in 2009/2010 to 506,456 patients in 2019/2020), significantly contributing to building up healthcare access pressure and challenges to deliver timely assessment and diagnosis^3^. Moreover, for non-urgent referrals for suspected basal cell carcinoma (BCC), the current waiting time is 18 weeks and only 80% of the patients were seen within this target time frame during 2019/2020^4^. Furthermore, COVID-19 contributed to an increased backlog of non-urgent cases due to cancellations or accommodating 2-week urgent patients, resulting in an estimated 17% shift to a later stage of melanoma in Europe during the post-lockdown period^5^. Skin cancer referrals are expected to increase in the coming years due to the ageing population in the UK^6^. Therefore, there is a need to develop new methods that can be used as a decision aid for the classification of suspicious or non-suspicious skin lesions during teledermatology triage.

The 7-point checklist (7PCL) method is recommended by the English National Institute for Health Care Excellence (NICE) to select patients with pigmented lesions and possible melanoma for urgent referral^7^. NICE also recommends the use of the ABCDE rule for identifying specific melanoma signs^8^. Another standard approach known as the Williams score^9^ is also used to estimate the lifetime risk of melanoma, the most lethal form of skin cancer. In recent years, there have been praiseworthy advancements in artificial intelligence (AI) technology, which proved to be cost-effective and secured their utilisation in support of healthcare applications. AI techniques have advanced the application of deep learning (DL)^10,11^ and vision-based attention models^12^ in skin cancer detection using image data. However, the majority of skin cancer classification research has focused on image data and DL models, with relatively little work being done on skin cancer detection using metadata alone. Moreover, the performance of the standard methods used is not satisfactory as reflected by their low sensitivity scores, such as the 7PCL (sensitivity $68\pm 2.10\%$) and the Williams score (sensitivity $67\pm 1.90\%$). In an attempt to fill the mentioned research gaps and to further improve skin cancer detection performance through utilising patient metadata alone, we devised an AI framework consisting of skin lesion metadata collection, identification of a new list of skin cancer risk factors, and proposal of a new risk score. The AI framework ensembles five AI models for skin lesion classification into suspicious and non-suspicious classes. The new list of seven skin cancer risk factors identified by the AI framework outperforms the 7PCL and Williams risk factors in suspicious skin lesion detection and can be fused with the 7PCL and Williams risk factors, leading to significantly higher sensitivity ($85.24\pm 2.20\%$). This research work has made the following major contributions:

1. Collection of metadata of 53,601 skin lesions from 25,105 patients across a national network of private UK skin diagnostic clinics.
2. Identification of a new list of risk factors named “*C4C risk factors*” from a pool of 22 meta-features responsible for the development of all skin cancer subtypes (melanoma, SCC, BCC) through an ensemble of five AI models, which significantly outperforms the existing 7PCL and Williams methods with a balanced accuracy of $71.27\pm 1.10\%$ and sensitivity of $80.46\pm 2.50\%$.
3. Proposal of a new skin cancer risk score named “*C4C risk score*”, which is based on the weighting of “*C4C risk factors*” with weights determined by intelligent data analysis. Using the C4C risk score alone achieves 68.90% balanced accuracy and $76.09\pm 1.20\%$ sensitivity in classifying suspicious and non-suspicious skin lesions, significantly higher than the exiting 7PCL risk score and Williams risk score.
4. Fusion of the “*C4C risk factors*” with the 7PCL and Williams risk factors to find the best feature combination, which achieves the highest overall performance with a balanced accuracy of $73.18\pm 2.10\%$ and a sensitivity of $85.24\pm 2.20\%$.

## Seminal works

Due to significant advancements in AI technology, researchers now use dermoscopic images for skin cancer detection^10^. AI-based early skin cancer detection is an active area of research and has achieved the state of the art performance^13^. However, skin cancer detection solely based on patient metadata has been hardly explored. This study aims to unlock the potential of patient metadata in skin cancer detection and hence our literature survey is limited to those that only utilised patient metadata. In clinical settings, skin cancer was usually diagnosed using standard techniques such as the ABCDE rule^8^ and the 7PCL method^14^ until computer vision techniques were used in skin cancer diagnosis. The ABCDE checklist is used for assessing cancer-like characteristics such as lesion shape, asymmetry, border irregularity, colour variegation, lesion diameter, and lesion evolution over time. The 7PCL method considers seven risk factors, i.e., change of lesion size, shape, colour, lesion > 7 mm, inflamed, oozing, and itching, to identify patients with features suspicious of melanoma and to recommend urgent referral. On the other hand, the Williams method^9^ is based on a validated scoring system that includes seven risk factors: patient age, gender, sunburn history, natural hair colour, density of freckles on arms, number of moles, and prior non-melanoma history. It can be pointed out that both the 7PCL and Williams methods consider a limited set of risk factors to calculate the respective risk scores. In the literature, we found only the 7PCL and Williams scoring methods are purely based on patient metadata for suspicious skin lesion detection and focus on suspicious melanoma. To the best of our knowledge, there is limited published work on the extensive use of patient metadata for detecting all skin cancer subtypes.

In recent years, open-source resources such as the International Skin Imaging Collaboration (ISIC) datasets and DL-based models have facilitated assessing skin lesion images for skin cancer detection. Esteva et al.^15^ compared the performance of DL models versus 21 board-certified dermatologists, indicating that DL models achieved a dermatologist-level performance. Conversely, the study in^16^ emphasised the importance of patient’s clinical information such as age, gender and lesion location, and found an overall 7% increase in balanced accuracy by the inclusion of clinical information in the analysis. Similarly, the work in^17^ evaluated all combinations of dermoscopic, macroscopic, and clinical metadata (age, gender, and anatomic location) and observed that combining all three achieved the highest overall AUC of 88.80%. The study in^18^ evaluated the use of the clinical information alone (age, gender, BMI, ethnicity, hypertension, heart disease, and diabetes status), which was based on the National Health Interview Survey (NHIS) data from 450,000 patients between 1997 and 2015, to classify non-melanoma skin cancers against the “never-cancer” skin diseases. They employed a basic feed-forward neural network and achieved an AUC of 81%, with a sensitivity of 86.2% and a specificity of 62.7%.

The studies in^16–18^ included a limited set of metadata (age, gender, and anatomic location). However, these studies used metadata along with image data, and there is no mention of the performance of their models using metadata alone.

## Data and methods

### Metadata collection

We collected and analysed the metadata of 53,601 skin lesions from 25,105 patients who attended Check4Cancer (C4C)’s private skin cancer diagnosis clinics in the UK between 2015 and 2022. The patients were informed that collected data might be used for research and the data was de-identified to ensure confidentiality. Informed consent was obtained from the patients. Following the approval from the University of Essex Research Ethics Committee on 8th February 2023 (Ref. No: ETH2223-0619), anonymised clinical metadata were transferred to C4C’s server for AI model development. C4C is a private healthcare company registered in the UK that provides cancer screening and diagnostic services for skin cancer patients. C4C has permission to use the collected metadata and holds ISO 27001 and Cyber Essentials certification, as they are accustomed to handling personal and medical (special category) data. C4C is fully compliant with UK data protection legislation and the duty of confidentiality and has followed the guidelines provided with the ethical approval.

This was a multi-centre study, however, clinical data was collected according to protocol by centrally trained nurses, with central reporting of all skin lesions by a central team of skin cancer specialists. All patients who attended the skin cancer diagnosis clinic from 2015 to 2022 were included in the study. The lesions were eligible to be assessed if they are: (1) located in adults > 18 years, (2) between 1 and 3 suspicious lesions which are not larger than the dermatoscopic lens (< 15 mm). For each lesion, we included 23 meta-features, as shown in Table 1: 7PCL (lesion size, lesion shape, lesion colour, lesion > 7 mm, lesion inflamed, lesion oozing, and lesion itching), lesion score based on the 7PCL, Williams risk factors (patient age, patient gender, hair colour, moles, sunburn, freckles, prior non-melanoma skin cancer), the overall Williams score, Williams group, prior melanoma, prior family history of skin cancer, lesion location, lesion age and whether this was a predominantly non-pigmented pink lesion. All the features except for lesion rating were considered candidate features for identifying the new risk factor sets to classify skin lesions into suspicious versus non-suspicious categories.

**Table 1:** List of 23 clinical meta-features: a total of 53,601 skin lesions metadata from 25,105 patients were collected.

S.no.	Meta-feature	Description	Type	Range
1	Lesion size	Change in size (yes/no)	Categorical	0–1
2	Lesion shape	Change in shape (yes/no)	Categorical	0–1
3	Lesion colour	Change in colour (yes/no)	Categorical	0–1
4	Lesion > 7 mm	Diameter 7mm or more? (yes/no)	Categorical	0–1
5	Lesion inflamed	Is it inflamed? (yes/no)	Categorical	0–1
6	Lesion oozing	Is it oozing? (yes/no)	Categorical	0–1
7	Lesion itching	Is it itchy? (yes/no)	Categorical	0–1
8	Lesion score	7-point weighted checklist score based on^14^	Numeric	0–10
9	Age	Patients’ age in years	Numeric	0–92
10	Gender	Patients’ gender at birth (M/F)	Categorical	0–1
11	Hair Colour	Natural hair colour (black, red, blonde, brown)	Categorical	1–4
12	Mole	Number of moles (1, 2, 3 or more, none)	Categorical	1–4
13	Sunburn	Number of sunburns (0, 1–4, 5–9, > 10 burns)	Categorical	1–4
14	Freckle	The density of freckles on arms (a few, several, a lot, none)	Categorical	1–4
15	Prior skin cancer	Any prior history of non-melanoma skin cancer (yes/no)	Categorical	0–1
16	Williams score	Williams score calculated based on^9^	Numeric	0–67
17	Williams group	Williams group (< 25 $=$ average risk; 25$+=$ high risk)	Categorical	0–1
18	Prior melanoma	Any prior history of melanoma (yes/no)	Categorical	0–1
19	Prior family history	Prior family history of skin cancer (yes/no)	Categorical	0–1
20	Lesion location	Location on body- Head Neck, Hand, Foot, .	Categorical	0–6
21	Lesion age	Has it been present < 6 months? (yes/no)	Categorical	0–1
22	Lesion pink	It is pink? (yes/no)	Categorical	0–1
23	Lesion rating	Target variable whether lesion is suspicious or non-suspicious	Categorical	0–1

The meta-features listed in Table 1 are self-explanatory except for the lesion location feature that comprises seven values according to the anatomic location of the lesion, such as (1) Head and Neck, (2) Trunk waist up (front or back), (3) Groin/Buttocks/Genitals, (4) Hand, (5) Foot, (6) Left/Right Leg (ankle up), and (7) Left/Right Arm (wrist up). The Williams score is calculated based on the method explained in the study^9^ and summarised in Table 2, where age, gender, sunburns, natural hair colour, the density of freckles on arms, number of moles, and prior non-melanoma history features are included to estimate the final Williams score. The lesion score is estimated based on a weighted 7PCL as mentioned in the study^14^ using the following equation:

1 $$\begin{aligned} \mathrm {Lesion~Score} = 2\sum _{i=1}^3 M_{i} + \sum _{j=1}^4 N_{i} \end{aligned}$$

where *M* is a set of major lesion features (lesion size, lesion shape, lesion colour) and *N* is a set of minor features (lesion>7mm, lesion inflamed, lesion oozing, lesion itching).

**Table 2:** Calculation of Williams score based on the risk factors described in the study^9^.

Risk factor	Category	Score
Gender	Female	0
	Male	7
Age	35–44	0
	45–54	5
	55–64	8
	65–74	11
Sunburn	None	0
	1–4	1
	5–9	4
	10 or more	7
Hair colour	Dark brown/Black	0
	Light brown	4
	Blond	5
	Red	8
Freckle	None	0
	Few	4
	Several	6
	A lot	10
Mole	None	0
	1	3
	2	5
	3 or more	11
Prior Skin Cancer	No	0
	Yes	13

The 7PCL was first formulated by Mackie et al.^19^. They used seven equally weighted lesion characteristics (change in size, shape, colour, inflammation, oozing, itching, and diameter $\ge$ 7 mm) to prioritise pigmented skin lesions for urgent referral. Walter et al.^14^ achieved better results with a revised version, which separated lesion features into two groups: (1) major features (change in size, shape, and colour), each having a weight of 2, and (2) minor features (inflammation, oozing, itching, and diameter $\ge$ 7 mm) with a weight of 1, as shown in Eq. (1). Consequently, lesions with a lesion score $\ge$ 3 were sent for a specialist opinion. Finally, the target variable, with a lesion rating as suspicious or non-suspicious, was assessed by the in-house skin cancer specialists. The experts classified pigmented lesions with atypical features in size, shape, colour, or dermatoscopic appearance of melanoma as suspicious. Furthermore, skin lesions suspicious of either BCC, SCC or potentially pre-malignant Actinic Keratoses were also rated as suspicious. Biopsy results were also available. However, we have a limited number of lesions that went for biopsy (only 10% of lesions undergo biopsy). If we use biopsy results as the target variable, the data size will reduce significantly (90%). As a result, the lesion rating was used here as the target variable rather than the biopsy results. The ultimate goal is to use AI as a clinical decision aid for the classification of suspicious or non-suspicious skin lesions during teledermatology triage.

### Statistical data analysis

Patient metadata consists of meta-features with categorical/text values, such as patient gender-taking values of male and female. There was a need to convert these categorical values into numerical as most AI models work well with numerical data rather than categorical data. We encoded all the non-numerical meta-features to convert them into numerical features using a one-hot encoding approach as summarised in Table 3 for an illustrative purpose.

**Fig. 1:**
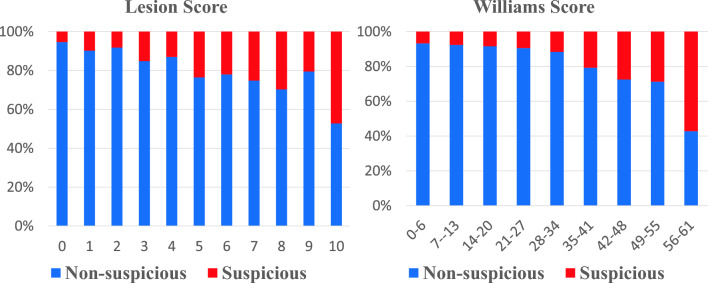
Comparison of lesion score and Williams score for suspicious and non-suspicious cases.

An effort was made to analyse the collected meta-features through explanatory data analysis. We examined all the meta-features and highlighted the findings using lesion pink and age risk factors for illustrative purposes. First, we analysed the lesion score meta-feature (range 0–10) using a bar plot and showed a difference between suspicious and non-suspicious cases using a statistical *t* test. Almost 50% of the lesions with a lesion score of 10 belong to the suspicious category. In contrast, only 5% of lesions with a score of 0 belong to the suspicious group, as highlighted in Fig. 1. Therefore, it can be inferred that lesion score and outcome variable, i.e., lesion rating, are highly correlated and the probability of a skin lesion being suspicious is likely to increase for higher values of the lesion score (*p* value $< 0.01$). For a Williams score between 56 and 61, around 60% of cases belong to the suspicious category, whereas only around 7% of cases belong to the suspicious group with Williams scores between 0–6, as shown using a bar plot in Fig. 1. It is highly likely that the higher the Williams score the higher the chance that the skin lesion is suspicious.

**Table 3:** Metadata.

Raw metadata before pre-processing				Metadata after pre-processing		
Lesion shape	Gender	Lesion rating		Lesion shape	Gender	Lesion rating
Yes	F	Green	$\Longrightarrow$	1	0	0
Yes	M	Green		1	1	0
No	F	Green		0	0	0
Yes	F	Red		1	0	1
Yes	M	Red		1	1	1
No	M	Green		0	1	0
Yes	M	Red		1	1	1
Yes	M	Green		1	1	0
No	F	Red		0	0	1
Yes	F	Red		1	0	1

**Fig. 2:**
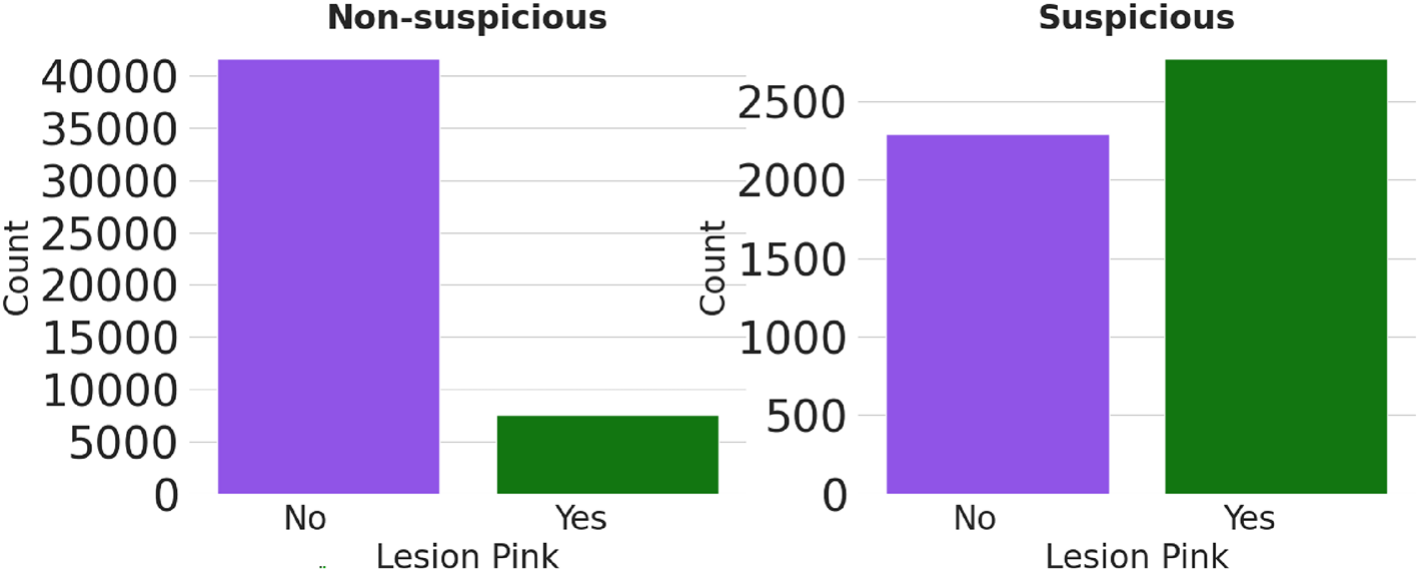
Comparison of meta-feature lesion pink for suspicious and non-suspicious cases.

**Fig. 3:**
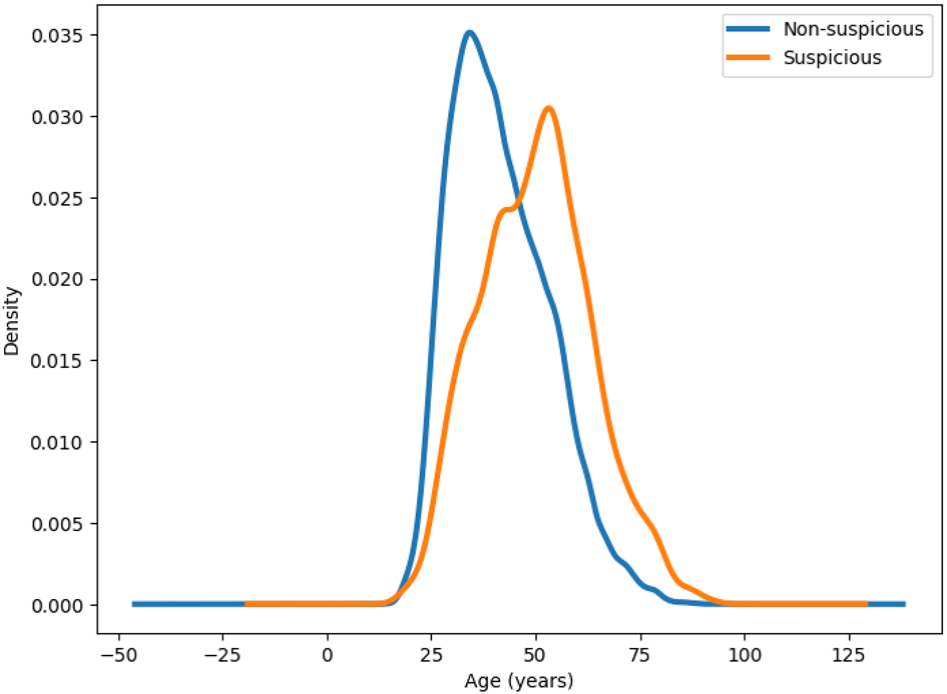
Comparison of patient age distributions for suspicious and non-suspicious cases.

We analysed another meta-feature of potential importance: “lesion pink”. Its statistics is shown using a bar plot in Fig. 2. We observed that about 83% of skin lesions with lesion pink value ‘no’ belong to the non-suspicious group as compared to only about 17% skin lesions with lesion pink value ‘yes’. Therefore, it can be ascertained that there is a low probability of being a suspicious lesion if the lesion pink value is ‘no’. Conversely, there is a comparatively higher chance (54%) for a skin lesion to be suspicious if it is a pink lesion, as shown in Fig. 2. Another meta-feature we analysed is “patient age”. Its distributions for suspicious and non-suspicious cases are compared in Fig. 3. We noted that the average age of patients with suspicious skin lesions is 52, markedly higher than the average age of 41 for patients with non-suspicious skin lesions (*p* value $<0.01$).

### Identification of new risk factors

**Fig. 4:**
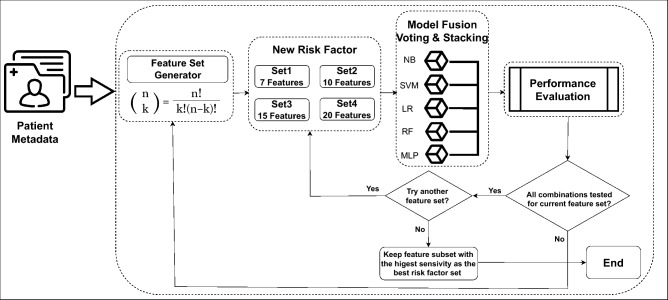
The proposed AI framework to identify a set of new risk factors for skin lesion classification.

For achieving reliable results, we adopted an ensemble of five machine learning (ML) models for identifying a new set of effective risk factors from a pool of 22 skin lesion meta-features, the first 22 attributes as shown in Table 1. An overview of the proposed AI framework for identifying a new set of risk factors for skin lesion classification into suspicious or non-suspicious class is shown in Fig. 4. The motivation behind classifying skin lesions into suspicious versus non-suspicious categories instead of traditional melanoma versus benign classes has emerged from the fact that early suspicious skin lesion detection could substantially increase 5-year survival rates by 20%. We used the combination formula: $$\left( {\begin{array}{c}n\\ k\end{array}}\right) = ^{n}C_{k}=\frac{n!}{k!(n-k)!}$$ for feature subset generation and proposed to generate four potential feature subsets of different sizes: Set1, Set2, Set3, and Set4, with 7, 10, 15, and 20 meta-features, respectively. Firstly, by applying combination theory we generated various combinations of 7 meta-features out of 22, resulting in a total of 170,544 combinations for Set1. We repeated feature subsets generation using the combination theory for Set2, Set3, and Set4, respectively. The best meta-feature combinations for these four sets were selected based on their overall performances of the ML models.

#### Naive Bayes (NB) classifier

This is a candid and compelling model for the classification task based on the Bayes theorem^20^. The class with the highest probability is considered as the predicted class for the given data tuple. NB classifiers assume that all attributes are conditionally independent of the given class label. The goal of this classifier is to learn a representative function from a given labelled training dataset. The conditional probability *p*(*Y*|*X*) of the target variable *Y* is calculated as follows:

2 $$\begin{aligned} { p(Y\mid {X} )={\frac{p(Y)\ p(X \mid Y)}{p( {X} )}}\,} \end{aligned}$$

where *p*(*Y*) is the prior probability of class *Y*, *p*(*X*|*Y*) is the conditional probability of data *X* given a particular class, and *p*(*X*) is the evidence or probability of data *X* regardless of its target class (suspicious or non-suspicious in our study).

#### Support vector machine (SVM)

This is one of the most popular supervised ML models, more frequently used for classification in various industries such as healthcare applications^21^. SVM finds a hyperplane to maximise the margin between the groups by utilising the Lagrangian optimisation technique^22^. One of the fundamental advantages of SVM is that if the data is linearly separable, then there is a unique global maximum value of the margin. In cases of non-linear distribution of the data, where a hyperplane cannot separate the data, SVM uses a kernel function that transforms the data into a higher dimensional feature space where the data’s linear separation is possible.

#### Logistic regression (LR)

This is a statistical method, in which log-odds of the probability of an event are linear combinations of independent variables^23^. Although the model outputs the probability of an event, it can be used for the classification task by applying a threshold. The logistic regression approach’s outcome is binary, such as positive or 1 (suspicious) and negative or 0 (non-suspicious). In our study, the LR was implemented to represent a relationship (function) between the meta-features and outcome variables by finding the best descriptive fitting model. Two different approaches were available for learning this function. A discriminating model learns the function directly to compute class posterior while a generative model learns the conditional class probability and class prior by applying the Bayes rule^24^. We used a modified alternative to discriminative and generative models to merge probability altogether to learn the discriminative function, which directly maps meta-feature input to output target variable as follows:

3 $$\begin{aligned} p(Y|X) = {\frac{exp(\beta _0 + \sum _{i=1}^P \beta _ix_i )}{1 + exp (\beta _0 + \sum _{i=1}^P \beta _ix_i)}} \end{aligned}$$

where *p*(*Y*|*X*) is the probability of a skin lesion being suspicious ($Y=1$) given a meta-feature vector *X*, $\beta _0$ is the intercept, $\beta _1,..., \beta _P$ are the coefficients, and *P* is the total number of meta-features.

#### Random forest

It employs an ensembling technique that generates multiple random decision trees and combines the outcomes of the decision trees given a test sample based on majority voting or averaging^25^. In our study, the decision trees were built upon a bootstrap sample of the data. RF adds more randomness in selecting a subset of predictors compared to a standard decision tree, where each node is split using the best variable selected based on a node splitting criterion—gini or entropy. This randomness in selecting features makes the RF classifier more accurate and robust compared to other classifiers such as SVM, discriminative analysis, and neural networks^26^. In our experiment, the RF model was optimised by finding the best hyper-parameters (number of trees, 500, max depth, 40, splitting criterion, gini, bootstrap, true) for classifying skin lesions into suspicious and non-suspicious categories.

#### Multi-layer perceptron (MLP)

This is one of the dominant predictive models used in ML applications^27^. MLP consists of an input and output layer and a hidden layer (in most cases) to transform input into some form of internal representation that the next layer can use. An MLP helps find a pattern or feature extraction from data that is considered complicated or laborious for a human. The success of neural network approaches such as MLP is due to a technique known as “backpropagation”, which allows changing the weights of the hidden layer if there are any errors. The fundamental advantage of MLP is that it does not require in-depth knowledge about the relationship between meta-feature input and output target variables. Instead, it tries to recognise patterns in the dataset and store those patterns in the form of weights for later use for the test cases. In our implementation, an MLP with three hidden layers (32, 16, 8 neurons) was adopted, with a rectified linear activation function (ReLU), and trained using the adaptive moment estimation (Adam) optimizer.

In this study, majority voting was adopted for decision-making by combining the outcomes of NB, LR, SVM, RF, and MLP. In the stacking approach, we stacked NB, LR, SVM, and RF as feature extractors and MLP as meta-learners to classify input metadata into suspicious and non-suspicious classes.

### A new skin cancer risk score

**Fig. 5:**
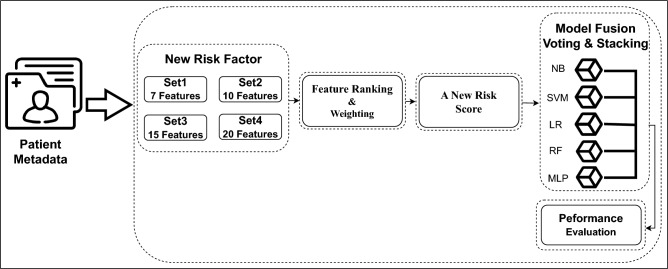
An overview of the proposed AI framework using patient metadata to identify a set of new risk factors followed by ranking and weighting those risk factors to deduce a new risk score for skin lesion classification.

An overview of the proposed AI framework for deriving a new skin cancer risk score for suspicious versus non-suspicious skin lesion detection is illustrated in Fig. 5. We used an LR model to rank the identified new risk factors. Feature ranking helps to find the most important features and provides an interpretation of the AI model on why certain features are more important than others and therefore can discard those features having the lowest/no correlation with the outcome variables. Furthermore, it can facilitate the reduction of the data collection burden in the future (e.g., instead of collecting 23 meta-features, data collection can be reduced to 7 meta-features only), as well as reduce model complexity and training time.

In our experiment, a set of *N* training samples were used, with each sample represented in the form of (*X*, *Y*), where *X* is a meta-feature vector and *Y* is the corresponding output (i.e., the target class). A classification rule is formulated using the training data to assign a class label, suspicious ($Y_{test}=1$) or non-suspicious ($Y_{test}=0$), to a new test input $X_{test}$, by minimising the probability of error. Typically, a feature is deemed relevant if it aids in distinguishing between classes and is not redundant with other relevant features. As shown in Eq. (3), the LR model produces posterior probabilities through a linear function of elements in the meta-feature vector *X*, ensuring that the probabilities sum to one and remain within the range of [0, 1]. The LR comes with a set of diagnostic tools that allows us to quantify the goodness-of-fit of the proposed model and select the features accordingly. The performance of the model is evaluated based on the maximum value of the log-likelihood (LL) achieved for each feature from *X* using the deviance D defined below:

4 $$\begin{aligned} D \,=\, -2 (\hbox {LL of the current model} \,-\, \hbox {LL of the saturated model}). \end{aligned}$$

The saturated model is the one with the number of parameters equal to the sample size, the likelihood of which is one. Low deviance values indicate a good fit, or equivalently, a high predictive value of the corresponding features. The deviance is useful for comparing two models with different numbers of features. The reduction in the deviance by adding a new feature is identical to the likelihood-ratio statistic, which has a Chi-squared ($\chi ^2$) distribution, provided that the sample size *N* is large^23^. Hence, we can use the likelihood-ratio test to include the features sequentially in a forward-selection procedure. If the difference in the deviance of the models before and after adding a new feature is at or above the critical value, then the new feature is significant in predicting the target class, otherwise not.

Although we have investigated four new sets of risk factors where Set1, Set2, Set3, and Set4 consist of 7, 10, 15, and 20 risk factors, respectively, we wanted to benchmark the AI models’ performance using our proposed C4C risk score along with the 7PCL-based lesion score and Williams score. Therefore, to develop the C4C risk score, we only used the 7 risk factors from Set1. We believe that the use of 7 risk factors to develop our proposed C4C risk score facilitates us to do a fair comparison with the 7PCL-based lesion score (included 7 risk factors), and Williams score (included 7 risk factors). The 7 risk factors from Set1 were ranked based on their coefficient values obtained using the LR model. The highest-ranked risk factor was assigned the highest weight, and consequently, lower-ranked risk factors were assigned lower weights to calculate the weighted sum of the seven risk factors, leading to our proposed C4C risk score.

### Data split and evaluation metrics

The metadata for 53,601 skin lesions was divided into training and test datasets, comprising 80% and 20% of the data, respectively. To prevent data leakage during the split, all metadata corresponding to each patient was exclusively assigned to either the training or testing dataset. During the training, a tenfold cross-validation (CV) method was employed to construct the models. These models were optimised by adjusting hyperparameters, and the most effective models were chosen based on their tenfold CV results using the training data. Subsequently, the selected models were assessed using the test dataset. Using a tenfold CV for model development on the training dataset only while keeping the test dataset completely independent from model development reduces the risk of overfitting. The performance of the developed AI framework was assessed using the following evaluation metrics:

5 $$\begin{aligned} & \mathrm {Sensitivity~(Sen)} = \frac{TP}{TP + FN} \end{aligned}$$

6 $$\begin{aligned} & \mathrm {Specificity~(Spc)} = \frac{TN}{FP + TN} \end{aligned}$$

7 $$\begin{aligned} & \mathrm {Balanced~Accuracy~(Bal.~ Acc.)} = \frac{Sen + Spc}{2} \end{aligned}$$

8 $$\begin{aligned} & \mathrm {\text {AUC}} = p(Score(TP) > Score(TN)) \end{aligned}$$

where *TP*, *TN*, *FP*, *FN* refer to true positive (suspicious classified as suspicious), true negative (non-suspicious classified as non-suspicious), false positive (non-suspicious misclassified as suspicious), and false negative (suspicious misclassified as non-suspicious) instances, respectively. The area under the curve (AUC) of a classifier is the probability that a randomly chosen TP case will be ranked higher than a randomly chosen TN case.

## Results and discussion

Firstly, by applying combination theory we generated various combinations of 7 meta-features out of 22, resulting in a total of 170,544 combinations. Among these, the 7 meta-features listed as Set 1 in Table 4 exhibited the highest balanced accuracy and sensitivity, which are lesion colour, size, shape, inflammation, natural hair colour, lesion age, and pinkness. Similarly, we replicated the experiment for identifying risk factor sets 2, 3, and 4. Table 4 shows the best meta-features in sets 2, 3, and 4, comprising 10, 15, and 20 features, respectively. The performances of the identified sets of risk factors for skin lesion classification (suspicious vs. non-suspicious) were evaluated by training and testing the developed ML models. The performances of the four sets of skin cancer risk factors are also presented in Table 4. Using risk factor set1 with 7 meta-features only, the best-ensembled ML model achieved a balanced accuracy of $71.27\pm 1.10\%$, a sensitivity of $80.46\pm 2.50\%$, and a specificity of $62.09\pm 1.90\%$. The balanced accuracy and sensitivity were notably improved to 73.01% and 84.51%, respectively, when the risk factor set3 was used. However, using the larger risk factor set4 with further meta-features added did not enhance the model’s performance, as shown in Table 4.

**Table 4:** The list of new risk factor sets identified through applying AI-based model fusion.

Risk factor Set1	Risk factor Set2	Risk factor Set3	Risk factor Set4
Lesion Colour	Lesion Colour	Lesion Colour	Lesion Colour
Lesion Size	Lesion: Inflamed Lesion: Oozing	Lesion: > 7 mm	Lesion Size
Lesion Shape	Lesion: Itch	lesion: Inflamed	Lesion Shape
Lesion Inflamed	Prior Melanoma	Lesion: Oozing	Lesion: > 7 mm
Hair Colour	Family History	Lesion: Itch	Lesion: Inflamed
Lesion Age	Lesion Age	Patient Gender	Lesion: Oozing
Lesion Pink	Lesion Pink	Moles	Lesion: Itch
	Prior Skin Cancer	Freckles	Patient Gender
	Patient Gender	Prior Melanoma	Moles
		Family History	Freckles
		Williams Group	Sunburn
		Lesion Age	Hair Colour
		Lesion Pink	Williams Group
		Lesion Body	Prior Melanoma
		Patient Age	Family History
			Williams Risk Group
			Lesion Age
			Lesion Pink
			Lesion Body
			Patient Age
Bal. Acc.: 71.27%	Bal. Acc.: 71.51%	Bal. Acc.: 73.01%	Bal. Acc.: 72.18%
Sensitivity: **80.46%**	Sensitivity: 80.67%	Sensitivity: **84.51%**	Sensitivity: 84.20%
Specificity: 62.09%	Specificity: 62.36%	Specificity: 61.51%	Specificity: 60.16%
AUC: 70.13%	AUC: 71.30%	AUC: 72.95%	AUC: 72.16%

Significant values are in bold.

An attempt was made to benchmark the newly proposed C4C risk factors with the 7PCL and Williams risk factors. The performance of the C4C risk factors for skin lesion classification into suspicious or not suspicious is presented and compared with that of the 7PCL and Williams risk factors in Table 5 where we showed that our approach outperformed the 7PCL and Williams method in terms of balanced accuracy and sensitivity scores (*p* value $<0.01$). It is interesting to note that lesion age, lesion pink and hair colour are risk factors for all skin cancer subtypes, which are not among the 7PCL since it is supposed to be only relevant for melanoma, the pigmented type of skin cancer. Finally, we evaluated the performance gain from feature fusion, with results also shown in Table 5. It is noteworthy that the highest performance with a balanced accuracy of $73.18\pm 2.10\%$, a sensitivity of $85.24\pm 2.20\%$ and a specificity of $61.12\pm 0.90\%$ was achieved when the C4C risk factors were fused with the 7PCL and Williams risk factors, which forms a set of 18 meta-features. We investigated the performance gain for each feature from the 7PCL and Williams risk factors. The feature subset generator as mentioned in Sect. 3 was used to find the optimal feature set, which shortlisted 11 external risk factors (patient age, patient gender, Williams score, Williams group, sunburn, freckles, moles, lesion body, lesion itch, lesion > 7 mm, and lesion oozing). Fusing them with the C4C risk factors can achieve better performance with the developed ML models, but it is at the price of collecting much more metadata. Finally, the performance of the C4C risk score is compared with that of the 7PCL lesion score and Williams score in Table 6. The C4C risk score alone achieved a sensitivity of $76.09\pm 1.20\%$ and a specificity of $61.71\pm 0.6\%$, significantly outperforming the 7PCL-based risk score (sensitivity $73.91\pm 1.10\%$, specificity $49.49\pm 0.50\%$) and Williams risk score (sensitivity 60.$68\pm 2.10\%$, specificity $60.87\pm 0.80\%$).

**Table 5:** Performance gain comparison through fusing the 7PCL, Williams and C4C risk factors.

Method	Risk factor	Sensitivity	specificity	Bal. Acc.	AUC
7PCL	1. Lesion Size, 2. Lesion Colour,	$68.09\%$	$61.07\pm 0.90\%$	$64.58\%$	$64.20\%$
	3. Lesion Shape, 4. Lesion > 7mm,				
	5. Lesion Inflamed, 6. Lesion Oozing,				
	7. Lesion Itch				
Williams	1. Patient Gender, 2. Patient Age,	$66.32\%$	$61.71\%$	$64.01\%$	$66.23\%$
	3. Sunburn, 4. Hair Colour,				
	5. Moles, 6. Freckles,				
	7. Prior Skin Cancer				
C4C	1. Lesion Colour, 2. Lesion Shape,	80.46%	62.09%	71.27%	70.13%
	3. Lesion Size, 4. Lesion Inflamed,				
	5. Hair Colour, 6. Lesion Age,				
	7. Lesion Pink				
Fusion:	1. Lesion Size, 2. Lesion Colour,	79.10%	59.42%	69.76%	70.45%
7PCL	3. Lesion Shape, 4. Lesion >7mm,				
Williams	5. Lesion Inflamed, 6. Lesion Oozing,				
	7. Lesion Itch, 8. Patient Gender,				
	9. Patient Age, 10. Sunburn,				
	11. Hair Colour,				
	12. Moles, 13. Freckles,				
	14. Prior Skin Cancer				
Fusion:	1. Lesion Size, 2. Lesion Colour,	**85.24%**	61.12%	**73.18%**	74.15%
7PCL	3. Lesion Shape, 4. Lesion >7mm,				
Williams	5. Lesion Inflamed,				
C4C	6. Lesion Oozing,				
	7. Lesion Itch, 8. Lesion Pink,				
	9. Lesion Age, 10. Patient Age,				
	11. Patient Gender, 12. Lesion Body,				
	13. Moles, 14. Williams Score,				
	15. Sunburn, 16. Williams Group,				
	17. Hair Colour, 18. Freckles				

Significant values are in bold.

**Table 6:** Performance comparison of the new *C4C risk score* with the 7PCL-based lesion score and Williams score.

Method	Risk score	Sensitivity	Specificity	Bal. acc.	AUC
7PCL	Lesion score based on Eq. 1	$73.91\pm 1.10\%$	$49.49\pm 0.50\%$	$61.70\pm 1.10\%$	$60\pm 0.90\%$
Williams	Williams score based on Table 2	60.$68\pm 2.10\%$	$60.87\pm 0.80\%$	$60.49\pm 0.90\%$	$62.50\pm 1.3\%$
C4C	C4C risk score	**76.09 ± 1.20%**	$61.71\pm 0.6\%$	**68.90±0.80%**	69.70 ± 1.20%

Significant values are in bold.

It is noted that the study in^16^ included patient metadata such as patient age, gender, lesion location, lesion bleeding, and lesion pain along with patient skin images and they reported a 7% performance improvement due to the addition of metadata to image assessment. However, they did not report the contribution of metadata alone in detecting skin cancer. Another study^28^, which won the Kaggle 2020 melanoma challenge, included more limited patient metadata, such as age, gender and lesion location, and observed that combining metadata along with lesion images did not improve their model performance.

To the best of our knowledge, the majority of the previous studies used image data only and there is limited work done on using patient metadata to classify lesions for skin cancer detection. Therefore, we have developed an AI framework solely based on metadata and observed that it can separate suspicious skin lesions from non-suspicious ones with a high sensitivity, which has the potential to support current skin cancer assessment when considered alongside image data. In the future, patients with both metadata and images classified as non-suspicious could be reassured without referral to a specialist clinic. Furthermore, the C4C risk score can be used as a decision-aid by telemedicine reporters to help with final lesion classification that is equivocal after image classification alone. This has the potential to reduce the number of referrals to a specialist clinic for possible biopsy and help reduce the waiting times for skin cancer diagnosis.

## Conclusion

Using AI techniques for skin lesion classification based solely on metadata has great potential to partly automate and facilitate the detection of suspicious lesions. With a reduction in patient referrals for possible biopsies, waiting times for skin cancer diagnosis and treatment will be shortened, resulting in improved outcomes. In this study, we developed an AI framework based on patient metadata for skin lesion classification, which outperformed the existing 7PCL and Williams methods. This study also contributed to high-quality data collection followed by the identification of a subset of meta-features highly relevant to skin cancer diagnosis. In our current and future research, we are extending our investigation through the fusion of the newly identified skin risk factors and weighted risk score together with lesion images using deep learning models, which we believe will further boost the performance of skin cancer detection in a cost-effective manner.

## Supplementary Information

Supplementary Information.

## Data Availability

The dataset collected and analysed during the current study is not publicly available yet as it contributes to a patent application that is currently underway. To query about the data from this study please contact our co-author Gordon Wishart (gcwishart@check4cancer.com).
