# Supplementary material for: Leveraging AI and patient metadata to develop a novel risk score for skin cancer detection
Source: Sci Rep. 2024 Sep 6;14:20842. doi: 10.1038/s41598-024-71244-2 (PMC11379912; doi:10.1038/s41598-024-71244-2)
Supplement: Supplementary file 1 — Supplementary Information. [file 41598_2024_71244_MOESM1_ESM.pdf]

Supplementary Documents

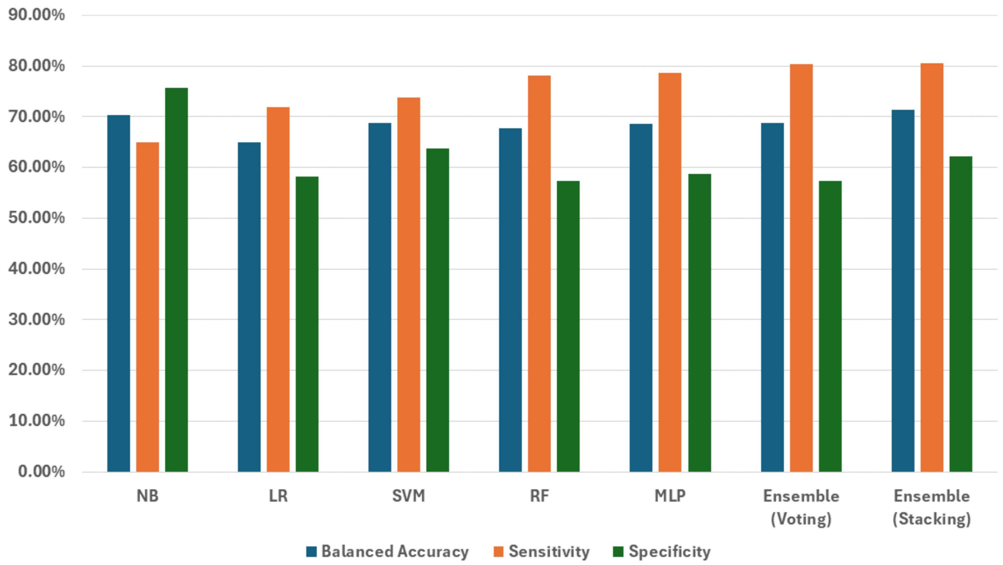

Figure 6. The bar-plot performance comparison of standalone and stacking models for skin lesion classification using the risk factor set1 (seven risk factors) listed in Table 4.

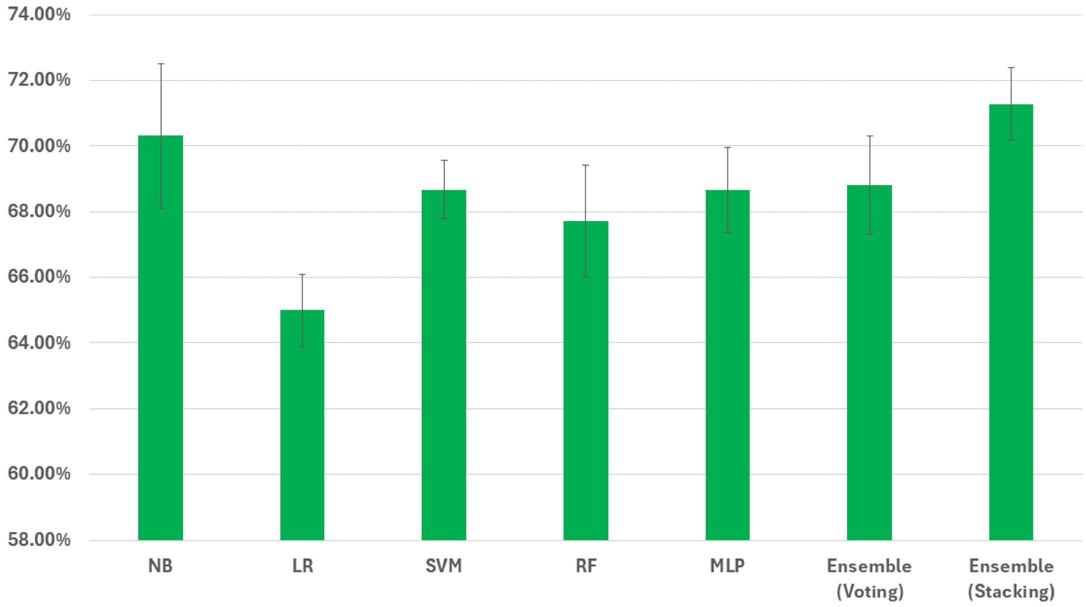

Figure 7. The box-plot of balanced accuracy performance comparison of standalone and stacking models for skin lesion classification using the risk factor set1 (seven risk factors) listed in Table 4.

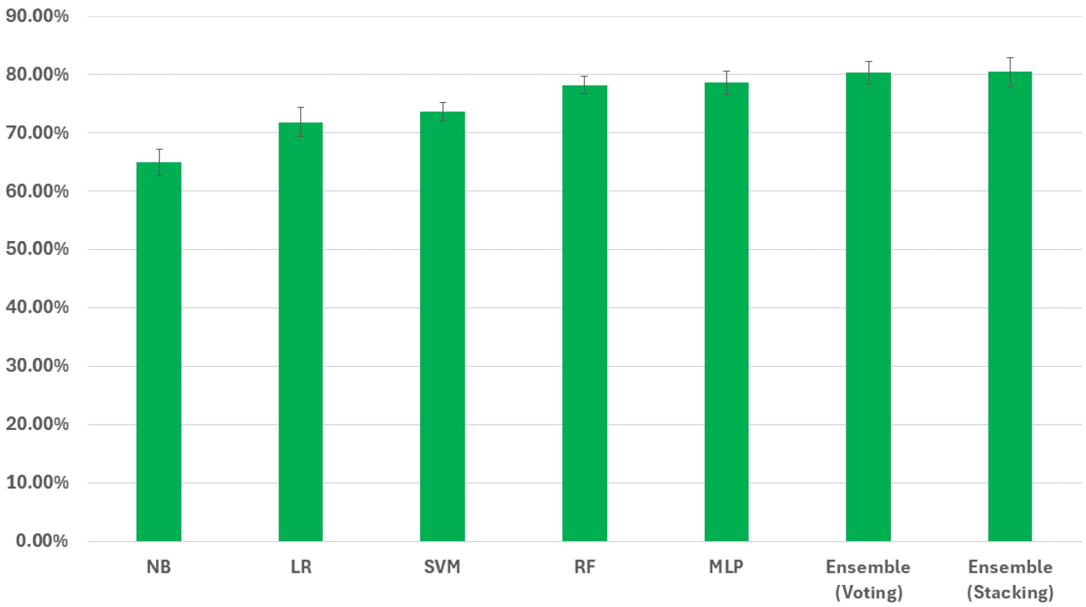

Figure 8. The box-plot of sensitivity performance comparison of standalone and stacking models for skin lesion classification using the risk factor set1 (seven risk factors) listed in Table 4.
